# Identifying Rare Circumstances Preceding Female Firearm Suicides: Validating A Large Language Model Approach

**DOI:** 10.2196/49359

**Published:** 2023-10-17

**Authors:** Weipeng Zhou, Laura C Prater, Evan V Goldstein, Stephen J Mooney

**Affiliations:** 1 Department of Biomedical Informatics and Medical Education School of Medicine University of Washington Seattle, WA United States; 2 Department of Psychiatry and Behavioral Health University of Washington Seattle, WA United States; 3 Harborview Medical Center School of Medicine University of Washington Seattle, WA United States; 4 Department of Population Health Sciences University of Utah Salt Lake City, UT United States; 5 Department of Epidemiology School of Public Health University of Washington Seattle, WA United States

**Keywords:** female firearm suicide, large language model, document classification, suicide prevention, suicide, firearm suicide, machine learning, mental health for women, violent death, mental health, language models, women, female, depression, suicidal

## Abstract

**Background:**

Firearm suicide has been more prevalent among males, but age-adjusted female firearm suicide rates increased by 20% from 2010 to 2020, outpacing the rate increase among males by about 8 percentage points, and female firearm suicide may have different contributing circumstances. In the United States, the National Violent Death Reporting System (NVDRS) is a comprehensive source of data on violent deaths and includes unstructured incident narrative reports from coroners or medical examiners and law enforcement. Conventional natural language processing approaches have been used to identify common circumstances preceding female firearm suicide deaths but failed to identify rarer circumstances due to insufficient training data.

**Objective:**

This study aimed to leverage a large language model approach to identify infrequent circumstances preceding female firearm suicide in the unstructured coroners or medical examiners and law enforcement narrative reports available in the NVDRS.

**Methods:**

We used the narrative reports of 1462 female firearm suicide decedents in the NVDRS from 2014 to 2018. The reports were written in English. We coded 9 infrequent circumstances preceding female firearm suicides. We experimented with predicting those circumstances by leveraging a large language model approach in a yes/no question-answer format. We measured the prediction accuracy with *F*_1_-score (ranging from 0 to 1). *F*_1_-score is the harmonic mean of precision (positive predictive value) and recall (true positive rate or sensitivity).

**Results:**

Our large language model outperformed a conventional support vector machine–supervised machine learning approach by a wide margin. Compared to the support vector machine model, which had *F*_1_-scores less than 0.2 for most infrequent circumstances, our large language model approach achieved an *F*_1_-score of over 0.6 for 4 circumstances and 0.8 for 2 circumstances.

**Conclusions:**

The use of a large language model approach shows promise. Researchers interested in using natural language processing to identify infrequent circumstances in narrative report data may benefit from large language models.

## Introduction

Suicide is a leading cause of death in the United States. Suicide risk factors include physical and mental health disorders, substance use disorders, prior exposure to violence, and having a firearm at home [1-4]. Firearm suicide has been more prevalent among men, but age-adjusted female firearm suicide rates have increased by 20% from 2010 to 2020, outpacing the rate increase among males by about 8 percentage points [5]. However, relatively few studies have focused specifically on female firearm suicide instead focusing on samples in which males are overrepresented (eg, military veterans) [6,7]. More data are needed to identify circumstances surrounding female firearm systems, and a primary source of these data derives from unprocessed narrative reports in the National Violent Death Reporting System (NVDRS).

In a previous study [1], we found that conventional natural language processing (NLP) algorithms could successfully identify some relatively common circumstances preceding female firearm suicide, using coroners’ and medical examiners’ (CMEs’) and law enforcement (LE) narrative reports provided by the NVDRS. However, because reliably training a conventional NLP pipeline requires a sizeable training data set, the approach worked well only for the most common preceding circumstances.

Recently, large language models such as ChatGPT were found to perform well on tasks such as answering yes/no questions and document classification [8-10]. Large language models were developed on the basis of large corpora of data crawled from the web and can be used to solve machine learning tasks in a question-answer format. Moreover, these large language models do not rely on the task's data set size. In this study, we explored the value of a large language model approach by framing our coding task as a binary response for classification. Specifically, we tested a large language model approach to identify infrequent circumstances preceding female firearm suicide and compared this approach’s performance to that of traditional machine learning models.

## Methods

### Overview

ChatGPT is the state-of-the-art large language model, but we could not use it directly in this study. Our data contain protected information; hence, we could not upload them directly to ChatGPT. Instead, we used open-source large language model alternatives. We ran these models locally to protect potentially sensitive information in the data. These models are developed similarly and are competitive in certain areas compared to ChatGPT. In a benchmark evaluation of large language models’ ability in problem-solving [11], FLAN-T5 [12] and FLAN-UL2 [13] were found to be less accurate than ChatGPT for world knowledge understanding and programming but competitive in following complex instructions, comprehension and arithmetic, and causal reasoning. In preliminary studies, we experimented with multiple large language models (FLAN-T5 [12], FLAN-UL2 [13], and others) and found that FLAN-UL2 performed the best. We hence used FLAN-UL2 for our subsequent experiments. Developed by Google LLC, FLAN-UL2 is an open-source, 20 billion–parameter encoder-decoder model and is useful for zero-shot learning (ie, the model makes predictions directly without further training).

### Data Sets

We used the NVDRS Restricted Access Database of female firearm suicides from 2014 to 2018 [1]. The data set contained unstructured CME and LE narrative reports describing the circumstances leading up to the suicide deaths of 1462 females. The reports were written in English. We manually coded 9 infrequent circumstances (ie, labels) preceding the firearm suicide deaths following the instructions specified by Goldstein et al [1]: sleep problems, abusive relationships, custody issues, sexual violence, isolation or loneliness, substance abuse, dementia, bullying, and caregiver issues. All infrequent labels occurred in <5% of the cases. We have provided details regarding the circumstance distribution in Table S1 in Multimedia Appendix 1. We split the data set into training and test sets with a 0.5:0.5 ratio.

### Model Evaluation

A prompt is the input for the large language model and will guide it for generating outputs. For FLAN-UL2, we designed a prompt as a pair of a narrative report and a question. The narrative report is the text we input into a traditional machine learning model. The question varies depending on the circumstances we want to the model to code. For example, for the circumstance “bullying,” “Answer the following yes/no question: was the decedent experiencing bullying in-person or online? Answer:” is the question. The model will yield an output of “Yes” if “bullying” is mentioned in the narrative report; if not, “No” will be the output. The question was adapted from the definition of each label developed through a previously reported manual review process with minimal changes [1]. A complete list of questions and definitions for each label is included in Table S2 in Multimedia Appendix 1. As a baseline, we used a series of conventional support vector machine (SVM) models [14] trained to identify each circumstance. FLAN-UL2 was only applied on the test set. SVM models were trained on the training set and applied on the test set. We repeated all experiments 5 times with resampling of the training and test sets. We reported the average *F*_1_-score, which is the harmonic mean of the precision (positive predictive value) and recall (true positive rate or sensitivity). The *F*_1_-score measures the model’s accuracy considering the imbalance in the data set and ranges from 0 to 1.

### Ethical Considerations

This study did not require approval from the University of Washington institutional research board because these deidentified data on deceased persons were not considered human subjects research. We received ethical approval from the National Violent Death Reporting System Restricted Access Database review committee (request number #410).

## Results

**Figure 1:**
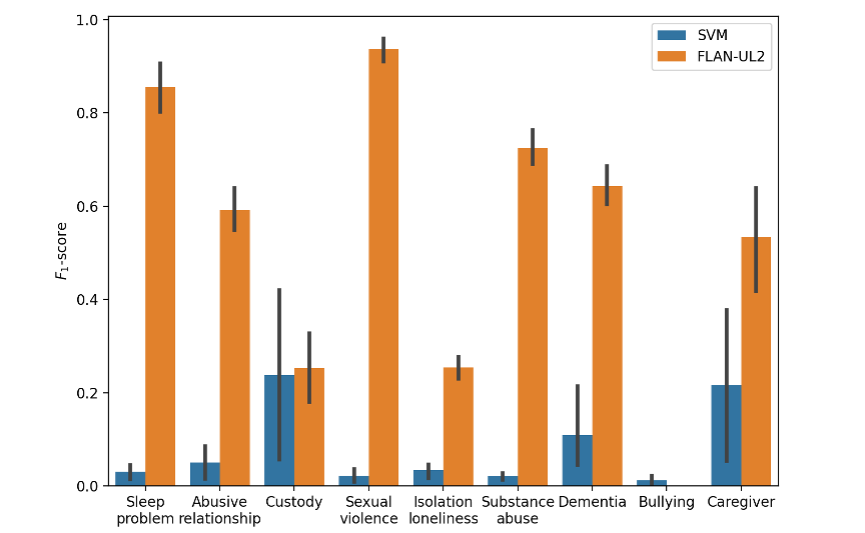
*F*_1_-scores of the support vector machine (SVM) and FLAN-UL2 model for coding rare circumstances of female firearm suicide from suicide reports when averaged over 5 runs. The height of the bars represents the mean *F*_1_-score, and the line at the tip of the bars represents the SD across 5 runs.

FLAN-UL2 performed better than the SVM for most of the female firearm suicide circumstances, sometimes substantially better (Figure 1). The *F*_1_-score of FLAN-UL2 was greater than 0.8 for “sleep problem” (3.4% prevalence) and “sexual violence” (2.6% prevalence). “Bullying” (1.6% prevalence) was the only circumstance where the SVM outperformed FLAN-UL2, and *F*_1_-scores were 0 or nearly 0 for all SVM and FLAN-UL2 runs. See Table S1 in Multimedia Appendix 1 for prevalence in other circumstances.

## Discussion

### Principal Findings

We found that the large language model (FLAN-UL2) outperformed the SVM for 8 of the 9 infrequent circumstances preceding female firearm suicide deaths. These findings suggest that a large language model approach can address a critical gap in identifying infrequent circumstances in unstructured text. Unlocking these data efficiently allows for subsequent analyses of female firearm suicide risk, including relationships among sexual violence, dementia, sleep problems, and firearm suicide.

The characterization of circumstances preceding female firearm suicides is an understudied area. In a previous study, Goldstein et al [1] used traditional NLP methods to predict 5 circumstances from suicide reports, with *F*_1_-scores ranging from 0.6 to 0.8. However, all these circumstances had a prevalence of at least 15%. In our study, all 9 circumstances had a prevalence of less than 5%. We complemented the existing work by providing a method for automatically coding circumstances preceding female firearm suicides at a larger scope.

The failure in identifying the “bullying” circumstance may be due to the fact that bullying is one of the most infrequent circumstances preceding female firearm suicide in the NVDRS. The question we provided to the large language model, “was the decedent experiencing bullying in-person or online?” might not be sufficiently sensitive for the model to understand how to identify these circumstances in the narrative reports. More detailed explanation of “bullying,” such as the victim was insulted or hurt at school or at the workplace, might be needed for the model to reason better. Large language models are known to be sensitive to prompt text, and designing an appropriate prompt (also known as prompt engineering) is an essential part of using large language models effectively [10]. Novel prompting techniques, such as few-shot learning (provide problem examples as part of the prompt) [10], have been proposed and may improve large language models’ performance. In this study, we used simple and consistent prompts to provide a baseline for using large language models to code infrequent circumstances preceding female suicide. Large language models are also computationally expensive. The experiments in this study were carried out on 2 NVIDIA A100 40 GB graphics processing units. Large language models are also known to be sensitive to “hallucination” [15], meaning that they generate paragraphs of texts that look reasonable but are factually incorrect. In this study, we prompted the model to generate yes/no answers, bypassing the risks of hallucination.

### Conclusions

Our large language model successfully identified infrequent circumstances preceding female firearm suicide deaths, having outperformed conventional NLP approaches by a wide margin. This finding suggests that large language models can be used to unlock textual analysis within public health research. More broadly, the success of our relatively simple queries at identifying infrequent circumstances suggests that large language models may be useful in public health surveillance, potentially allowing practitioners to track the prevalence of infrequent conditions that are never explicitly coded into surveillance systems. Future studies should explore the performance of different large language models and variations in the models’ underlying mechanisms when applied to coding infrequent circumstances.

## Appendix

Multimedia Appendix 1 Prevalence, definitions and derived questions of the rare female firearm suicide circumstances.

## Abbreviations

CME: coroners or medical examiners
LE: law enforcement
NVDRS: National Violent Death Reporting System
NLP: natural language processing
SVM: support vector machine
