# Supplementary material for: Identifying Rare Circumstances Preceding Female Firearm Suicides: Validating A Large Language Model Approach
Source: JMIR Ment Health. 2023 Oct 17;10:e49359. doi: 10.2196/49359 (PMC10618876; doi:10.2196/49359)
Supplement: Multimedia Appendix 1 [file mental_v10i1e49359_app1.docx]

**Table S1. Prevalence of the rare female firearm suicide circumstances.**

| **Circumstance** | **Count (%)** |
| --- | --- |
| Sleep problem | 49/1462 (3.4%) |
| Abusive relationship | 44/1462 (3.0%) |
| Custody | 43/1462 (2.9%) |
| Sexual violence | 38/1462 (2.6%) |
| Isolation loneliness | 37/1462 (2.5%) |
| Substance abuse | 32/1462 (2.2%) |
| Dementia | 26/1462 (1.8%) |
| Bullying | 23/1462 (1.6%) |
| Caregiver | 21/1462 (1.4%) |

**Table S2. Circumstance definitions and the questions adapted from them.**

| **Circumstance** | **Definition** | **Question** |
| --- | --- | --- |
| Sleep problem | The decedent was noted to be experiencing insomnia, sleep associated anxiety, sleep apnea, and/or trouble sleeping. | Was the decedent experiencing insomnia, sleep associated anxiety, sleep apnea, and/or trouble sleeping? |
| Abusive relationship | The decedent was noted to be providing care for a family member who required long-term assistance due to a chronic illness or physical limitation. | Was the decedent experiencing or recently got out of an intimate relationship that was described as abusive? |
| Custody issues | The decedent was noted to be having an ongoing legal child custody proceeding, had lost custody of their child, or have lost contact with their child. | Was the decedent having an ongoing legal child custody proceeding, had lost custody of their child, or have lost contact with their child? |
| Sexual violence | The decedent was noted have experienced sexual violence anytime in their lifetime. | Had the decedent experienced sexual violence anytime in their lifetime? |
| Isolation loneliness | The decedent was noted to not belong to any social environments or noted to be experiencing loneliness. | Did the decedent not belong to any social environments or was the decedent experiencing loneliness? |
| Substance abuse | The decedent was noted to have had a history of involvement with a substance use disorder treatment program. | Had the decedent had a history of involvement with a substance use disorder treatment program? |
| Dementia | The decedent was noted to be experiencing dementia or memory problems including diagnosed Alzheimer’s disease, Lewy Body dementia, and Parkinson’s disease. | Was the decedent experiencing dementia or memory problems including diagnosed Alzheimer's disease, Lewy Body dementia, and Parkinson's disease? |
| Bullying | The decedent was noted to be experiencing bullying in-person or online. | Was the decedent experiencing bullying in-person or online? |
| Caregiver issues | The decedent was noted to be providing care for a family member who required long-term assistance due to a chronic illness or physical limitation. | Was the decedent providing care for a family member who required long-term assistance due to a chronic illness or physical limitation? |
